# Supplementary material for: Deletion of wfs1 Impairs Oligodendrocyte Precursor Cells Dorsal Distribution and Myelination Through the wfs1-hmgcs1 Axis in Zebrafish
Source: Biology (Basel). 2026 Aug 16;15(16):1402. doi: 10.3390/biology15161402 (PMC13510151; doi:10.3390/biology15161402)
Supplement: Supplementary file 1 [file biology-15-01402-s001.zip › File S1-Transcriptome sequencing data and differentially expressed genes (DEGs) in wfs1-- zebrafish at 5 dpf/2.KEGG/wfs1KOvsWT/DOWN/src/dre03013.html]

dre03013


- dre:407710

- Down regulated genes

407710\_1(-1.82225070081538)
- dre:324342

- Down regulated genes

324342(-1.03757612952153)

- dre:393973

- Down regulated genes

393973\_1(-1.32009639670831)

- dre:100004503

- Down regulated genes

100004503\_1(-1.56223399709744)

- dre:100005893

- Down regulated genes

100005893\_1(-1.31383589537958)

- dre:393693

- Down regulated genes

393693(-1.24516760321911)

Close
